# Mitochondrial dynamics links PINCH-1 signaling to proline metabolic reprogramming and tumor growth

**DOI:** 10.15698/cst2021.02.241

**Published:** 2020-12-10

**Authors:** Ling Guo, Chuanyue Wu

**Affiliations:** 1 Guangdong Provincial Key Laboratory of Cell Microenvironment and Disease Research, Shenzhen Key Laboratory of Cell Microenvironment, Academy for Advanced Interdisciplinary Studies and Department of Biology, Southern University of Science and Technology, China.; 2 Department of Pathology, University of Pittsburgh School of Medicine, Pittsburgh, PA 15261, USA.

**Keywords:** tumor growth, fibrosis, mitochondrial dynamics, proline metabolism, cell-extracellular matrix adhesion, PINCH-1

## Abstract

Proline metabolism is critical for cellular response to microenvironmental stress in living organisms across different kingdoms, ranging from bacteria, plants to animals. In bacteria and plants, proline is known to accrue in response to osmotic and other stresses. In higher organisms such as human, proline metabolism plays important roles in physiology as well as pathological processes including cancer. The importance of proline metabolism in physiology and diseases lies in the fact that the products of proline metabolism are intimately involved in essential cellular processes including protein synthesis, energy production and redox signaling. A surge of protein synthesis in fast proliferating cancer cells, for example, results in markedly increased demand for proline. Proline synthesis is frequently unable to meet the demand in fast proliferating cancer cells. The inadequacy of proline or “proline vulnerability” in cancer may provide an opportunity for therapeutic control of cancer progression. To this end, it is important to understand the signaling mechanism through which proline synthesis is regulated. In a recent study (Guo *et al.*, Nat Commun 11(1):4913, doi: 10.1038/s41467-020-18753-6), we have identified PINCH-1, a component of cell-extracellular matrix (ECM) adhesions, as an important regulator of proline synthesis and cancer progression.

How does PINCH-1 regulate proline synthesis? Proline is synthesized in cells through reduction of Δ^1^-pyrroline-5-carboxylate (P5C) with NAD(P)H as an electron donor, a reaction catalyzed by pyrroline-5-carboxylate reductase (PYCR). Three mammalian PYCR isoforms (i.e., PYCR1, 2 and L), each of which is encoded by a different gene, have been identified. Consistent with an important role of proline synthesis in cancer progression, PYCR1 is one of the most frequently overexpressed metabolic enzymes in cancer. In mammalian cells PYCR1 is primarily localized in the mitochondria. Our previous studies have found that a fraction of kindlin-2, an integrin-binding protein that is concentrated at cell-ECM adhesions, is translocated into mitochondria where it directly interacts with PYCR1. The interaction of kindlin-2 with PYCR1 prevents proteolytic degradation of the latter, resulting in increased PYCR1 level, proline synthesis and cell proliferation. Growth of cancerous tissues such as lung adenocarcinoma is often associated with increased synthesis of collagen matrix, in which nearly 25% of amino acids are derived from proline. Increased collagen matrix synthesis stiffens ECM, which in turn promotes kindlin-2 mitochondrial translocation and interaction with PYCR1, resulting in further up-regulation of PYCR1 level and proline synthesis in cancer. Thus, there appears to be a positive regulatory cycle through which proline synthesis, collagen matrix production and cancer progression are intrinsically linked. Using a mouse model of lung adenocarcinoma, we have shown that disruption of this positive regulatory cycle by ablation of kindlin-2 from lung adenocarcinoma inhibits proline synthesis, tumor fibrosis and growth.

The importance of kindlin-2 mitochondrial translocation and interaction with PYCR1 in regulation of proline synthesis and cancer progression begs the question of what signaling proteins regulate kindlin-2 mitochondrial translocation and interaction with PYCR1. In our recent study, we have shown that PINCH-1 functions as a key regulator of these processes. Depletion of PINCH-1 markedly inhibits kindlin-2 mitochondrial translocation and interaction with PYCR1, resulting in down-regulation of PYCR1 level, proline synthesis and cell proliferation.

How does PINCH-1 regulate kindlin-2 mitochondrial translocation and interaction with PYCR1? Our recent studies reveal that PINCH-1 regulates these processes through control of mitochondrial fragmentation. Mitochondria are dynamic subcellular organelles that are constantly undergoing fission and fusion, the balance of which controls mitochondrial morphology and functions. Mitochondrial fission is mediated by dynamin-related *protein* 1 (Drp1) and its receptors. Loss of PINCH-1 markedly increased Drp1 expression, resulting in excessive mitochondrial fragmentation. Knockdown of Drp1 reverses PINCH-1-deficiency-induced defects in mitochondrial dynamics, kindlin-2 mitochondrial translocation and interaction with PYCR1, and restores PYCR1 level, proline synthesis and cell proliferation. Based on these findings, we propose a model in which PINCH-1 regulates kindlin-2 mitochondrial translocation, interaction with PYCR1, proline synthesis and cell proliferation through control of Drp1 expression and consequently mitochondrial fragmentation (**Fig. 1**). While excessive mitochondrial fragmentation inhibits kindlin-2 mitochondrial translocation and interaction with PYCR1 and consequently reduces PYCR1 level and proline synthesis, forced increase of proline synthesis (e.g., by overexpression of PYCR1) can reverse PINCH-1 deficiency-induced increase of Drp1 expression and excessive mitochondrial fragmentation. Thus, proline synthesis is not only regulated by mitochondrial dynamics but it can also impact mitochondrial dynamics. The signaling pathway through which proline synthesis regulates mitochondrial dynamics remains to be defined.

**Figure 1:**
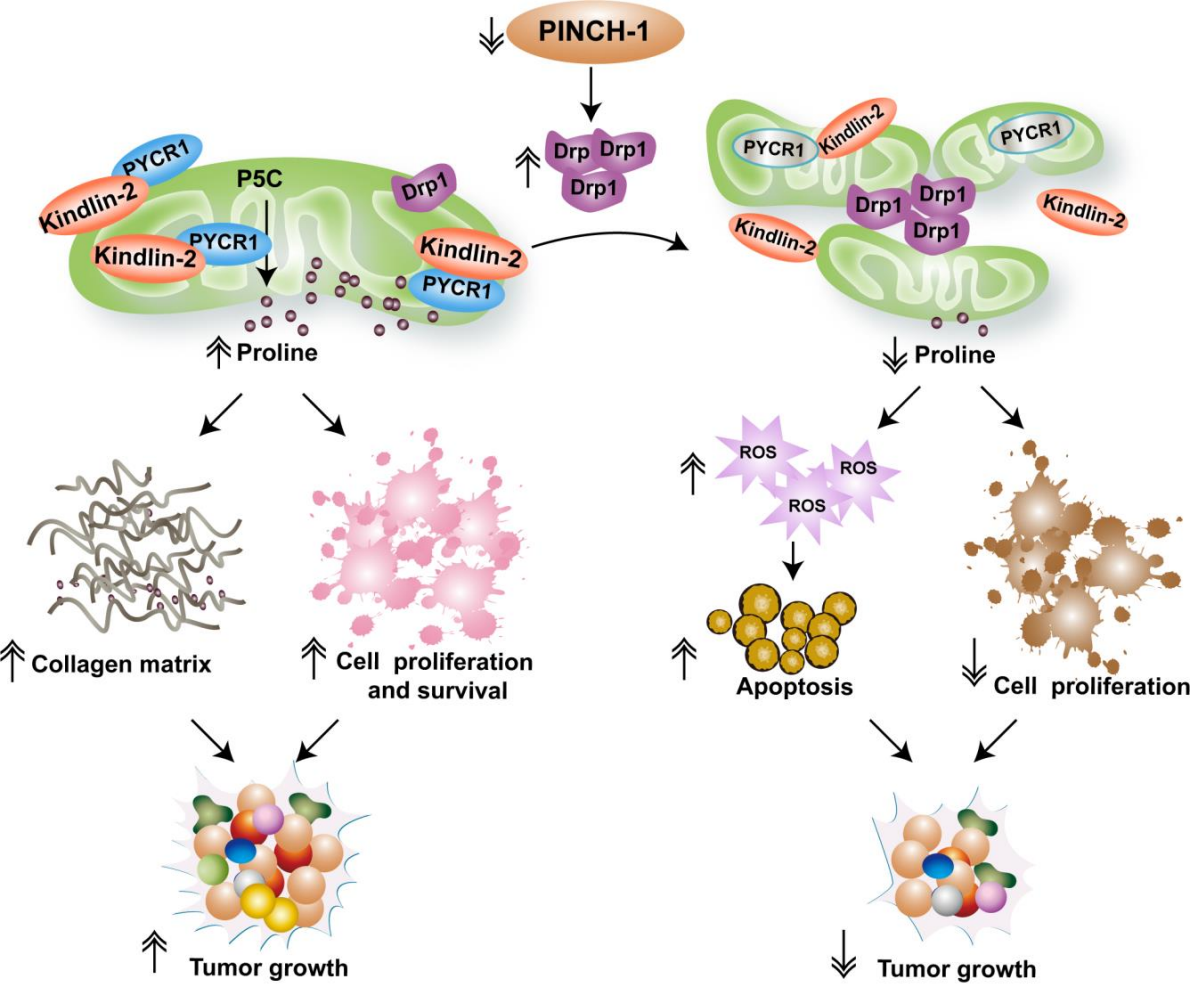
FIGURE 1: A model of PINCH-1-mediated regulation of mitochondrial dynamics and proline synthesis. The figure depicts a model in which mitochondrial dynamics links PINCH-1 signaling to proline metabolic reprogramming and tumor growth. Cells in cancerous tissues express an elevated level of PINCH-1, which suppresses Drp1 expression and prevents excessive mitochondrial fragmentation. PINCH-1 deficiency increases Drp1 expression, resulting in excessive mitochondrial fragmentation, which in turn inhibits kindlin-2 mitochondrial translocation and interaction with PYCR1. Inhibition of kindlin-2 interaction with PYCR1 promotes proteolytic degradation of PYCR1 and consequently reduces PYCR1 level and proline synthesis. Inadequacy of proline synthesis in cancer cells reduces cell proliferation, increases reactive oxygen species (ROS) signaling and promotes apoptosis. Furthermore, reduced proline synthesis in collagen matrix-producing cells (e.g., cancer-associated fibroblasts) restrict tumor-associated fibrosis. Collectively, these effects contribute to suppression of tumor fibrosis and growth in response to inhibition of PINCH-1 signaling.

Using cancerous tissues from human patients with lung adenocarcinoma as well as those from a mouse model of lung adenocarcinoma, we have shown that the level of PINCH-1 is markedly increased in lung adenocarcinoma. Consistent with a critical role of PINCH-1 in regulation of Drp1 expression and proline synthesis, ablation of PINCH-1 from lung adenocarcinoma in mouse markedly increases Drp1 expression and down-regulates PYCR1 level and proline synthesis, resulting in reduced cancer cell proliferation, collagen matrix production and tumor growth. The mortality rate of the mice with lung adenocarcinoma is significantly reduced in response to ablation of PINCH-1, underscoring the importance of PINCH-1 signaling in lung adenocarcinoma progression *in vivo*.

Proline metabolism influences not only protein synthesis and cell proliferation but also other processes such as redox balance and cell survival. Consistent with this, we have found that depletion of PINCH-1 from lung adenocarcinoma cells significantly reduces the NADPH/NADP^+^ ratio, increases reactive oxygen species (ROS) level and promotes apoptosis. Furthermore, PINCH-1 is known to participate in integrin-mediated cell-ECM adhesion and signaling. Thus, PINCH-1 deficiency-induced suppression of cancer progression likely reflects combined effects on multiple processes including inhibition of proline synthesis, collagen matrix production, cell proliferation and survival (**Fig. 1**). Alterations of cell-ECM adhesion, mitochondrial dynamics and metabolic activities are common features of cancer development and progression. The findings that a cell-ECM adhesion protein PINCH-1 regulates mitochondrial dynamics and proline metabolism illustrate a signaling mechanism through which these common features of cancer are linked. Targeting the PINCH-1 signaling pathway, therefore, may provide an attractive strategy for therapeutic control of cancer progression.

